# Mesenchymal Stromal Cells Engage Complement and Complement Receptor Bearing Innate Effector Cells to Modulate Immune Responses

**DOI:** 10.1371/journal.pone.0021703

**Published:** 2011-07-01

**Authors:** Guido Moll, Regina Jitschin, Lena von Bahr, Ida Rasmusson-Duprez, Berit Sundberg, Lena Lönnies, Graciela Elgue, Kristina Nilsson-Ekdahl, Dimitrios Mougiakakos, John D. Lambris, Olle Ringdén, Katarina Le Blanc, Bo Nilsson

**Affiliations:** 1 Department of Laboratory Medicine, Clinical Immunology and Transfusion Medicine, Karolinska Institutet and Hematology Center at Karolinska University Hospital Huddinge, Stockholm, Sweden; 2 Department of Immunology, Genetics and Pathology, Rudbeck Laboratory, Uppsala University, Uppsala, Sweden; 3 Department of Pathology and Laboratory Medicine, University of Pennsylvania School of Medicine, Philadelphia, Pennsylvania, United States of America; 4 Department of Oncology and Pathology, Cancer Center Karolinska (CCK), Karolinska Institute, Stockholm, Sweden; Albert Einstein Institute for Research and Education, Brazil

## Abstract

Infusion of human third-party mesenchymal stromal cells (MSCs) appears to be a promising therapy for acute graft-versus-host disease (aGvHD). To date, little is known about how MSCs interact with the body's innate immune system after clinical infusion. This study shows, that exposure of MSCs to blood type ABO-matched human blood activates the complement system, which triggers complement-mediated lymphoid and myeloid effector cell activation in blood. We found deposition of complement component C3-derived fragments iC3b and C3dg on MSCs and fluid-phase generation of the chemotactic anaphylatoxins C3a and C5a. MSCs bound low amounts of immunoglobulins and lacked expression of complement regulatory proteins MCP (CD46) and DAF (CD55), but were protected from complement lysis via expression of protectin (CD59). Cell-surface-opsonization and anaphylatoxin-formation triggered complement receptor 3 (CD11b/CD18)-mediated effector cell activation in blood. The complement-activating properties of individual MSCs were furthermore correlated with their potency to inhibit PBMC-proliferation *in vitro*, and both effector cell activation and the immunosuppressive effect could be blocked either by using complement inhibitor Compstatin or by depletion of CD14/CD11b-high myeloid effector cells from mixed lymphocyte reactions. Our study demonstrates for the first time a major role of the complement system in governing the immunomodulatory activity of MSCs and elucidates how complement activation mediates the interaction with other immune cells.

## Introduction

Based on their immunomodulatory properties, mesenchymal stem or stromal cells (MSCs) are under investigation as treatment for acute graft-versus-host disease (aGvHD) and other types of hematopoietic stem cell transplantation-related disorders [1], [2], [3]. Their production of trophic factors also makes MSCs valuable candidates for many types of tissue repair applications [4], [5]. The unique potential of MSCs to provide therapeutic options for thus-far untreatable human diseases is overshadowed by their difficult handling [6]. It is not clear yet how to assess the therapeutic potency of these cells before clinical administration to individual donors. Thus, it becomes clear that a more thorough mechanistic characterization of the most effective cell therapy product is urgently needed [5].

The exact mechanism by which MSCs elicit the broad immunomodulatory effect *in vivo* is at present unclear [5]. Is has been observed that intravenous administration of MSCs promotes a beneficial effect on damaged tissues by inhibiting apoptosis, stimulating cell regeneration, and increasing angiogenesis [5], [7]. It appears that MSCs reprogram recipient immune cells [8], [9], [10], for generating a complex immunosuppressive milieu consisting of a multitude of factors with complementary functions [6]. MSCs thereby synergize with the host's immune system to potently suppress acute immune responses, in a fashion similar to that described for the process of tumor immune modulation [11]. The complement system serves as an important signalling system for modifying immune responses [12], e.g., in modulating the anti-tumor immune response [13], [14]. Complement integrates the interaction between innate and adaptive immunity, it may be a key mediator of the broad immune modulation elicited by the therapeutic application of these cells, and it may possibly contribute to the generation of the immunosuppressive environment [14]. It has recently been suggested that complement anaphylatoxins C3a and C5a participate in activation and recruitment of MSCs to sites of tissue damage and repair [15].

MSCs, like many other cell therapy treatments, can be applied via intravenous infusion into the blood circulation. These treatments are generally characterized by a high rate of cell loss [16]. This may be due to the instant blood-mediated inflammatory reaction (IBMIR) [17], which is characterized by a rapid destruction of the infused cells due to complement-, coagulation- and platelet activation. Complement rapidly reacts against foreign pathogens and cooperates with innate immune cells to clear these alien structures [18]. The central step in complement activation, regardless of the triggering event, is the proteolytic cleavage of complement component C3 (187 kDa) into C3b (177 kDa) and C3a (9 kDa) [19]. This cleavage reaction leads to disruption of the highly reactive internal thioester group and allows the subsequent covalent attachment of C3b to the triggering surface. C3b can then undergo a series of proteolytic cleavages to produce the surface-bound fragments iC3b and C3dg. These cell-bound fragments are ligands for immune cells bearing complement receptor type 1 (CR1; CD35), CR2 (CD21), CR3 (CD11b/CD18), and CR4 (CD11c/CD18); with CR3 being most prominent on monocytes, macrophages and NK-cells. Once complement activation occurs, the soluble anaphylatoxins C3a and C5a are released, which attract and activate leukocytes [12]. C5a-receptor signalling leads to up-regulation of CD11b on myeloid cells, to promote the interaction with its ligand iC3b [20]; this reaction can be blocked with a small cyclic C5a-receptor antagonist [21], or by inhibiting cleavage of C3 with the cyclic peptide Compstatin [22]. Recently, complement activation was identified to be a major requisite for tumor cell-induced myeloid suppressor cell-generation *in vivo*, next to other soluble mediators such as PGE_2_, VEGF, IL-6 and IL-1β [13], [14]; all well known to be engaged in MSC-mediated immune modulation and evasion [6].

The aim of the present study was to characterize the interaction of culture-expanded human MSCs with the human complement system, and to analyze the resulting effector cell responses in human blood, in order to optimize this immunomodulatory treatment, and to find ways for improving the therapeutical efficiency of this novel treatment.

## Materials and Methods

### Ethics statement, MSC recipients and clinical grade MSCs

Human MSCs were obtained from bone marrow aspirates following approval by the ethics committee and review board at Karolinska University Hospital Huddinge, Sweden. The expansion and characterization of MSCs was performed according to the guidelines of the MSC consortium of the European Blood and Marrow Transplantation Group (EBMT), and was approved by the Swedish Medical Products Agency, as previously described in detail [2]. MSCs were isolated and characterized as described previously [1], [23]. Donors and patients, or their legal guardians, gave written informed consent [1], and the study was approved by the Regional Ethics Review Board. All patients have previously been reported [1], [3]. Forty-two MSC recipients who underwent hematopoietic stem cell transplantation (HSCT) at the Karolinska University Hospital, Huddinge, Sweden and received treatment with MSC, between the years of 2003 and 2010 were included in the analysis. Patients received myeloablative (n = 26) or reduced intensity conditioning (n = 16) and GvHD prophylaxis, according to previously published procedures [1]. The indications for MSC administration were failure of standard treatment approaches for acute GvHD refractory to standard therapy in 28 patients, and tissue injury after HSCT (hemorrhagic cystitis and pneumomediastinum) in 14 patients. Patients received MSC-infusions from 3^rd^ party unrelated donors (n = 50), from haploidentical related donors (n = 11) and from HLA identical siblings (n = 3). Patients received MSCs from passage 1 to 4 (P1-4) in doses of approximately 1.0 to 3.0×10^6^ cells/kg. All MSC donors (n = 33) were considered healthy after assessment of medical history, physical examination, and serological screening for HIV and hepatitis viruses. For fast availability, most of the applied cells were stored in liquid nitrogen and freshly thawed for IV-infusion. The MSC suspensions were culture-negative for bacteria and fungi, and polymerase chain reaction negative for Mycoplasma pneumoniae [2].

### Isolation and culture of cells for experiments

To isolate MSCs, bone marrow mononuclear cells were separated over a gradient of Redigrad (GE Health Care, Uppsala, Sweden), washed and resuspended in DMEM low-glucose medium (DMEM-LG; Gibco, Paisley, UK), supplemented with 100 IU/ml penicillin, 0.1 mg/ml streptomycin, and 10% fetal calf serum (FCS; Gibco), and plated at 1.6×10^5^ cells/cm^2^. When the cultures neared confluence (>80%), the cells were detached by treatment with trypsin and EDTA (Invitrogen, Grand Island, NY) and replated/passaged at a density of 4.000 cells/cm^2^. HUVECs (Promocell, Heidelberg, Germany) were grown in endothelial cell growth medium (Promocell), supplemented with 100 IU/ml penicillin and 0.1 mg/ml streptomycin and replated at 10.000 cells/cm^2^. Cells for experiments were detached with trypsin, viability was assessed by trypan blue exclusion (generally >95%), and cell suspensions were adjusted to 1-2×10^6^ cells/ml.

### Blood and serum preparations

Fresh non-anticoagulated human blood was obtained from healthy volunteers who had given informed consent in accordance with the Helsinki Protocol and received no medication for at least 10 days. Complement-active normal human AB serum (NHS) was processed within 1 h of blood collection and stored at -70°C, to maintain the complement activity. In all experiments that made use of human serum, the final concentration of NHS or NHS/EDTA was 50% (v/v). Whole blood flow cytometry was performed with blood that had been anticoagulated by using 0.05 mg/ml lepirudin to (Refludan; Hoechst, Frankfurt am Main, Germany), a specific thrombin inhibitor that maintains the complement function in blood. To block complement function, blood or NHS was treated with cyclic Compstatin Ac-I[CV(1MeW)QDWGAHRC]T (1628Da) [22] or a inactive linear control peptide Ac-IAVVQDWGHHRAT (1532Da) and with C5aR antagonist AcF-[OPdChaWR] (896Da) [21] or its respective control peptide Phe-[Orn-Pro-dCha-Ala-D-Arg]. The inhibitors and the control peptides were produced in the laboratory of J. D. Lambris.

### Complement- and antibody-binding assays

The cells were mixed with an equal volume of NHS or NHS/EDTA in sterile polystyrene FACS tubes (BD, Franklin Lakes, NJ). Non-serum-treated or NHS/EDTA-treated cells were used as negative/washing controls, respectively. Incubation was carried out for 20 min at 37°C. Complement activity was stopped by adding EDTA (final concentration 10 mM). Supernatants were harvested after pelleting the cells by centrifugation at 900 g for 5 min, and frozen at -70°C. In experiments requiring the presence of Ca^2+^ (detection of MBL, C1q, and annexin-V), cells were prepared under non-chelating conditions with binding buffer (10 mM HEPES/NaOH, pH 7.4, with 140 mM NaCl and 2.5 mM CaCl_2_) from BD [24]. Pellet fractions to be analyzed by western blotting and FACS were collected after three washes with PBS/EDTA or binding buffer.

### Western blot analysis

SDS-PAGE was performed in a Mini-Protean 3 electrophoresis apparatus according to the supplier's instructions (Bio-Rad, Hercules, CA). Cells were prepared as described above (NHS or NHS/EDTA in FACS tubes for 20 min at 37°C). To remove unbound proteins, cell pellets were washed three times with 10 mM PBS/EDTA, resuspended with protease inhibitors (SigmaFast; Sigma-Aldrich Sweden AB) and incubated with 0.1 M methylamine (pH 9.0) for 1 h at 37°C, to disrupt the covalent linkage of C3 fragments to the cells. Proteins were solubilized with lysing buffer (1% SDS, 10 mM Tris-HCL pH 7.4, and protease inhibitors) and cell debris was pelleted at 13,000 g. Equal amounts of protein were separated on a 10% SDS-PAGE, electroblotted onto a PVDF membrane (Perkin-Elmer, Boston, MA), and probed with a 1:8000 dilution of peroxidase-labeled rabbit anti-human C3d-HRP and anti-C3c-HRP antibody (Dako, Glostrup, Denmark). Blots were developed using the enhanced chemo-luminescence detection kit (Western Lightning; Perkin-Elmer, Boston, MA). Purified C3b, iC3b, and C3d (1 µg/lane) were used as positive controls for immunodetection.

### Flow cytometry

#### A. Phenotypic characterization of MSCs

Cell suspensions were labeled with respective antibodies (Ab), washed and analyzed on a FACScan flow cytometer (BD Biosciences, San Jose, CA). Cell acquisition was performed in a forward/sideward scatter (FSC/SSC) dot plot, cell debris was excluded with FSC, and dead cells were identified with propidium iodide. Fluorescence signals from 10,000 – 25.000 events were counted, with detection of median fluorescence intensity (MFI), and analyzed with Summit v4.1 software (Dako, Fort Collins, CO). The relative fluorescence intensity (RFI) was calculated by dividing the MFI of serum-treated cells by the MFI of non-serum-treated cells. MSCs/ECs were labeled with the following mouse anti-human mAb's (all from BD): isotype controls IgG1-FITC, and IgG2a-PE; negative controls CD45-FITC, and CD14-PE; positive controls CD90-FITC, and CD105-PE (Ancell, Bayport, MN); and complement regulatory proteins CD46-FITC, CD55-FITC, and CD59-PE. The following rabbit anti-human polyclonal Ab's were used: C3c-FITC, C3d-FITC, IgG-FITC, IgM-FITC, and C1q-FITC (all from Dako, Glostrup, Denmark). Binding of mouse anti-human iC3b mAb,[25] and mouse anti-human MBL mAb (Hycult Biotechnology, Uden, NL) was detected with FITC-conjugated F(ab')_2_-fragments of polyclonal rabbit anti-mouse Ab (Dako).

#### B. Whole-blood analysis

Whole-blood flow cytometric analysis was performed according to Mollnes et al. [20]. Blood was distributed equally into surface-heparinized FACS tubes (Corline Systems, Uppsala, Sweden), and treated with PBS, EDTA, antagonists, or matched control peptides. The final concentrations of inhibitors in blood were as follows: EDTA, 10 mM; Compstatin or its control peptide, 60 µM; and C5aRA or its control peptide, 10 µM. The blood was split equally into two tubes for each condition and either MSCs or a similar volume of PBS was added (100 µl/ml). Different doses of MSCs were tested (0.1-1.0×10^6^ cells/ml). The samples were incubated at 37°C, and remaining complement activity was stopped after 40 min by the addition of EDTA. Sample aliquots of 100 µl blood were collected after gentle mixing, labeled for 20 min with 5 µl of antibody (anti-C3c-FITC, or CD11b-FITC and respective isotype controls), and lysed for 5 min by adding 2 ml FACS lysing solution (BD Biosciences). The lysed samples were centrifuged at 900 g for 5 min, the supernatants discarded and the cells were washed once again with 3 ml of PBS; 50,000 events were analyzed. The remaining blood sample volumes were diluted in an equal volume of 10 mM PBS/EDTA and centrifuged for 5 min at 2000 g to yield plasma supernatants for use in ELISA analysis. These supernatants were stored at -70°C until use.

### Mixed lymphocyte reactions

Mixed lymphocyte reactions (MLRs) were performed as described elsewhere [23]. Responder PBMCs were stimulated with either PHA-mitogen, or alloantigen-stimulated with a pool of allogeneic donors (n = 5), and irradiated third-party MSCs (P3-5) were added at a 1∶10 ratio to PBMCs. To block complement function different types of antagonists were added to MLRs: linear Compstatin, 20 µM; cyclic Compstatin, 20 µM; and cyclic C5aR-antagonist, 5 µM. MACS-depletion was used to remove the CD14/CD11b-high fraction from PBMCs (Miltenyi Biotech, Germany); additionally blocking experiments of complement receptor 3 (CD11b/CD18) were performed with anti-CD11b mAb (5 µg/ml; Acris Biotechnology, Germany) or respective isotype control IgG (5 µg/ml). MSC-mediated suppression of alloantigen-stimulated PBMC proliferation was assessed at day 5 to 6 with [^3^H]thymidine incorporation (18 h) as counts per minute (cpm).

### ELISA analysis

C3a and sC5b-9 generation in plasma supernatants was measured by ELISA according to the method of Nilsson Ekdahl et al. [26]. Values are expressed in ng/ml and AU/ml, respectively, if not otherwise indicated.

### Statistical analysis

Statistical analyses were performed using Student's t-test or ANOVA. If the data did not fit normal distribution the Mann-Whitney test or the Wilcoxon matched pairs test was used (two-tailed confidence intervals, 95%; P<0.05 was considered statistically significant; Prism 5.0; Graphpad Software).

## Results

### MSCs and ECs display differential complement activating and regulatory properties

**Figure 1:**
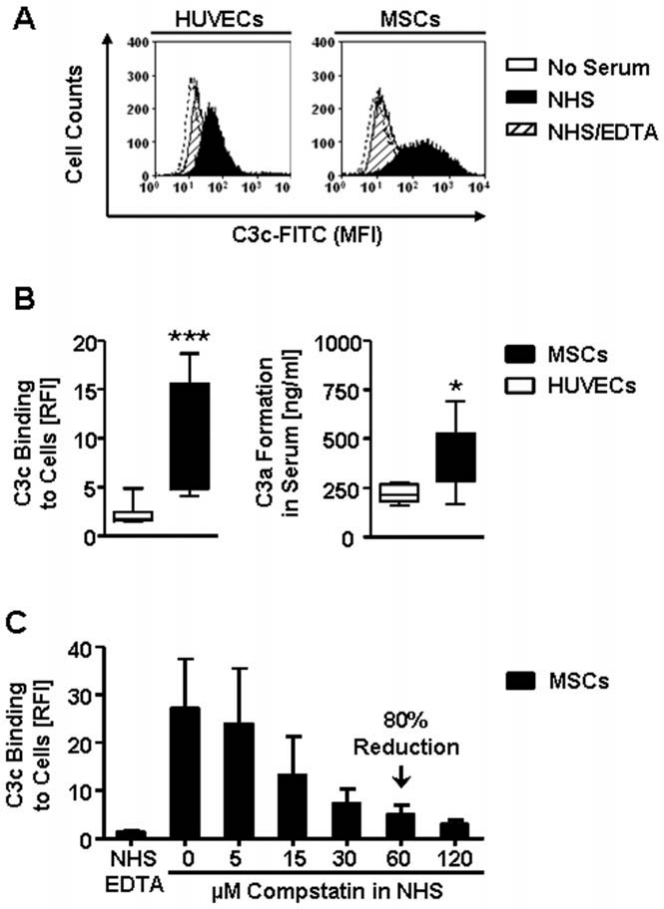
MSCs and ECs activate complement to different degrees. Freshly trypsinized MSCs (black) and HUVECs (white) were exposed to complement active normal human serum (NHS) in order to study the cell surface binding of complement activation products with flow cytometry. (**A**) Histogram overlays for binding of anti-C3c antibody to non-serum-treated cells (empty, dotted), NHS- (black), or NHS/EDTA-treated cells (shaded) is shown after a 20 min incubation with the respective sera at 37°C. Complement-inactivated NHS (NHS/EDTA) served as negative control. (**B**) Box plots (whiskers min/max) for anti-C3c binding (RFI, left panel, n = 11) and C3a generation in supernatants (ng/ml, right panel, n = 8) after incubation of cells with NHS, the relative fluorescence intensity (RFI) was calculated compared to non-serum-treated cells. The data are expressed as means±SEM, *P<0.05, and ***P<0.001. (**C**) Inhibition of C3 fragment binding after pre-treatment of NHS with different doses (5-120 µM) of specific C3-inhibitor Compstatin (n = 5).

Flow cytometry was used to characterize the binding of complement factor C3 fragments to MSCs and HUVECs after the cells were incubated with complement-active normal human serum (NHS). MSCs showed a signal shift for anti-C3c detection relative to non-serum-treated cells or HUVECs treated in a similar fashion (Fig. 1A). Binding of C3 fragments was only observed after incubation with complement-active NHS, but not after treatment with EDTA-inactivated NHS (NHS/EDTA), demonstrating that C3 fragments only bound when complement was active. Significantly higher levels of C3 fragments were bound to the cell surface of MSCs, as compared to HUVECs (P<0.001, Fig. 1B), which was accompanied by C3a formation in the supernatants (P<0.05). Activation and subsequent binding of C3 to the surface of MSCs could be prevented by pre-treatment of NHS with Compstatin in a dose-dependent manner (Fig. 1C). NHS-treatment did not result in increased propidium iodide-incorporation, Annexin-V-binding, or lysis of MSCs, as compared to NHS/EDTA treated cells (data not shown).

**Figure 2:**
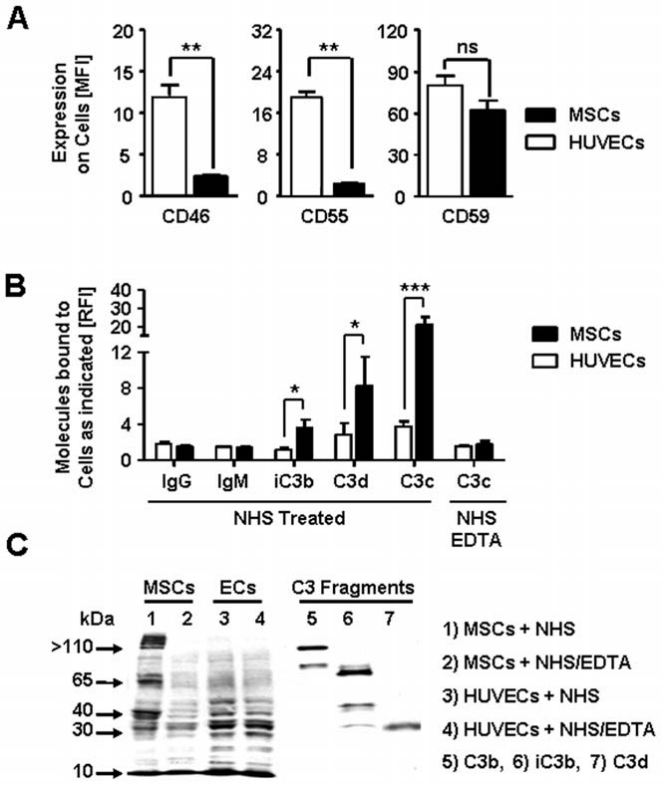
MSCs and ECs differ in their complement regulatory activity. (**A**) Expression of complement regulatory proteins MCP (CD46), DAF (CD55), and CD59 on MSCs and HUVECs (n = 11 each) was quantified with flow cytometry (MFI, median fluorescence intensity). (**B**) Cell surface binding of immunoglobulins (IgG and IgM) and specific C3 fragments (iC3b, C3d, C3c) was analyzed after cell treatment with NHS or NHS/EDTA and labeling with specific antibodies directed against the following epitopes: IgG (n = 8), or IgM (n = 8); iC3b (n = 5), C3d (n = 9), C3c (n = 15). **(C)** Representative western blot for detection of C3c, iC3b and C3d epitopes bound to NHS or NHS/EDTA treated cells. Purified C3b, iC3b and C3d served as positive controls. The data in figure A and B are means±SEM, with: *P<0.05, **P<0.01, ***P<0.001.

In order to study the intrinsic complement regulatory capacity of the two cell types, we analyzed the expression of complement regulatory proteins CD46, CD55, and CD59. Trypsin detached ECs strongly expressed all three regulatory molecules, whereas low to medium passage MSCs (P4-6) which had been harvested in a similar fashion expressed only CD59, but showed a relative lack for expression of CD46 and CD55 (both P<0.01, Fig. 2A), which suggests a substantial lack in complement regulatory capacity for culture expanded MSCs. We also studied the differential cell surface binding of complement activating factors (Fig. 2B). Both cell types bound small amounts of immunoglobulins, but no significant differences in binding of IgG, IgM, C1q, and MBL were found. The strongest positivity was found for the most part on necrotic cells, as identified by propidium iodide incorporation (data not shown).

In order to clarify the specific profile of C3 fragments bound to the MSC-surface we performed flow cytometric analysis with antibodies which have reactivity against the C3c-, iC3b- and C3d-epitopes within the C3 molecule. We found that MSCs bound higher amounts of all three fragments on their cell surface (P<0.05 and P<0.001, Fig. 2B), which did not occur with EDTA-inactivated NHS. The presence of iC3b on MSCs was furthermore suggested by western blot experiments using polyclonal anti-C3c and C3d antibodies (Fig. 2C, lane 1). Bands with strong reactivity for C3 epitopes were detected on NHS-treated, but not on NHS/EDTA-treated MSCs. These bands corresponded to the 63- and 40-kDa fragments of the beta- and alpha- chain of control iC3b, respectively. Higher molecular weight bands (>110 kDa) larger than the native C3 alpha-chain were also found. No iC3b was found on HUVECs. Most interestingly, freshly thawed MSCs (as prepared for clinical use) displayed much higher degrees of complement fragment binding than did freshly trypsinized cells (data not shown), which indicates that minor damage to the cell-surface integrity/polarity as a result of the freeze/thaw procedure might have affected their complement triggering/regulating properties.

### Complement-mediated modulation of MSC-induced effector cell priming in human blood

**Figure 3:**
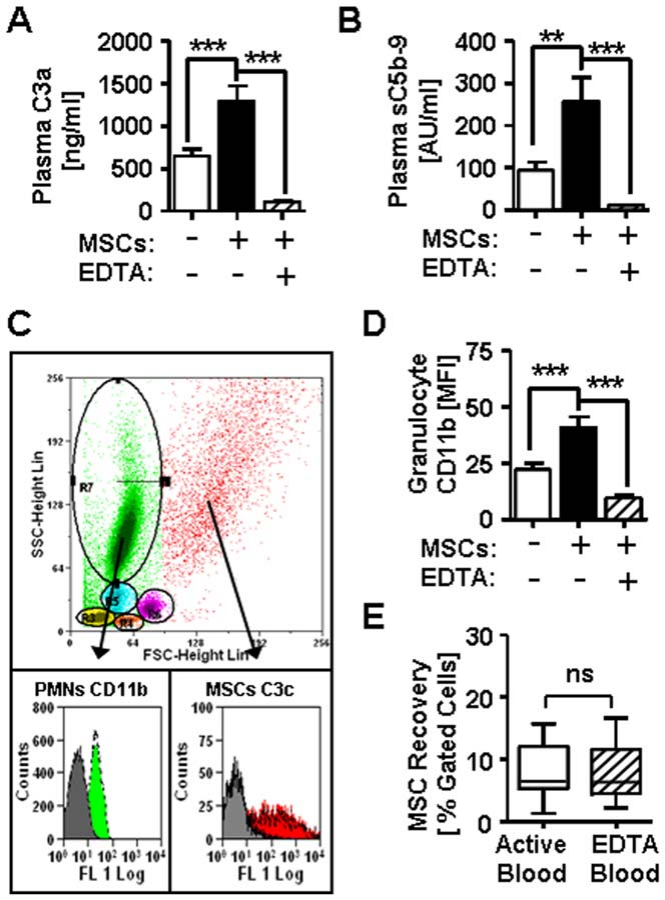
Blood exposure of MSCs activates complement and effector cells. Lepirudin-anticoagulated blood was incubated with MSCs (black), and blood treated with either PBS (white) or 10mM EDTA (shaded) served as active or inactive control, respectively. (**A**) Plasma levels of C3a (ng/ml, n = 13), and (**B**) Plasma level of soluble C5b-9 complex (AU/ml, n = 13) were detected with ELISA. (**C**) Flow cytometric analysis of PBMCs and MSCs after labeling of individual blood aliquots with specific antibodies and subsequent erythrocyte lysis; the cells were first gated according their scatter profile (top panel, scatter plot) and representative histograms for triggering of CD11b-expression on PMNs (green) or binding of C3-fragments to MSCs (red) are shown compared to EDTA-inactivated negative control blood (grey histograms). (**D**) Up-regulation of CD11b on PMNs in blood (MFI, n = 19). The median fluorescence intensity (MFI) of the cell-surface marker CD11b was assessed with flow cytometry. (**E**) Percentage of recovered MSCs after a 40 min exposure to active or EDTA-inactivated blood (n = 17). The data in figure A-E are means±SEM; with: ns = not significant; **P<0.01, and ***P<0.001.

**Figure 4:**
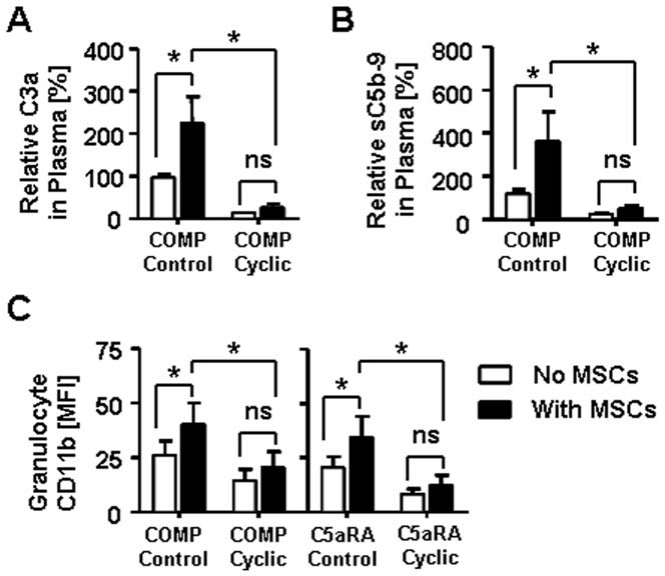
Effector cells activation in blood is mediated by complement activation products. Lepirudin-anticoagulated blood was pre-treated with either complement antagonist compstatin or a specific C5a receptor antagonist (C5aRA), and their respective control peptides, and then incubated for 40 min with either MSCs (black) or PBS (white) as negative control (n = 6). (**A**) Relative generation [%] of C3a and (**B**) sC5b-9 in blood after incubation w/wo MSCs in presence of cyclic compstatin or its linear control peptide (both 60 µM). The results are presented relative to active non-MSC-treated blood, which was set to be 100%. (**C**) Upregulation of CD11b on PMNs in blood treated w/wo MSCs in presence of cyclic compstatin or control peptide (60 µM), and C5aR-antagonist (C5aRA) or control peptide (10 µM). The data in figure in A-C are means±SEM, with *P<0.05.

Since clinical MSCs are applied via systemic infusion to our HSCT patients, we simulated the exposure of MSCs to lepirudin-anticoagulated ABO-compatible human blood *in vitro*. Lepirudin inhibits thrombin, but maintains complement activity in blood. In agreement with our prior findings in NHS, exposure of lepirudin-blood to MSCs revealed a significant increase in generation of complement activation product C3a (P<0.001, Fig. 3A) and soluble C5b-9 complex (P<0.01, Fig. 3B), which is indicative for formation of C5a. Flow cytometric analysis of MSCs and different blood effector cells (Fig. 3C), confirmed the activation and binding of C3 fragments to MSCs in blood (shown in red), which was accompanied by triggering of CD11b on neutrophil granulocytes (shown in green, P<0.001, Fig. 3D) and monocytes (data not shown). We furthermore analyzed the general activation of effector cells by detecting morphological changes with flow cytometry (Table S1), which revealed a significant reduction in the number of resting monocytes and lymphocytes (P<0.001 and P<0.01), and an increase in activated lymphocytes and monocytes (both P<0.01) in response to MSCs. But no significant changes in percentage of gated MSCs was found when active blood was compared to EDTA-inactivated control blood (8.1±4.4 vs. 7.3±4.6), which indicates that no immediate complement- or effector cell-mediated lysis of MSCs had taken place (Fig. 3E). To verify if the activation of effector cells after contact with MSCs is mediated via complement signalling we blocked complement function by pre-treatment of blood with Compstatin, which lead to strongly reduced formation of both C3a and sC5b-9 (P<0.05, Fig. 4A and 4B). Compstatin furthermore abrogated triggering of CD11b on effector cells (P<0.05, Fig. 4C, left panel), which could also be blocked with a specific C5a receptor antagonist (P<0.05, Fig. 4C, right panel). But neither formation of C3a, sC5b-9, or triggering of CD11b was affected by the corresponding control peptides.

### The complement-activating properties of MSCs affect their immunomodulatory profile

**Figure 5:**
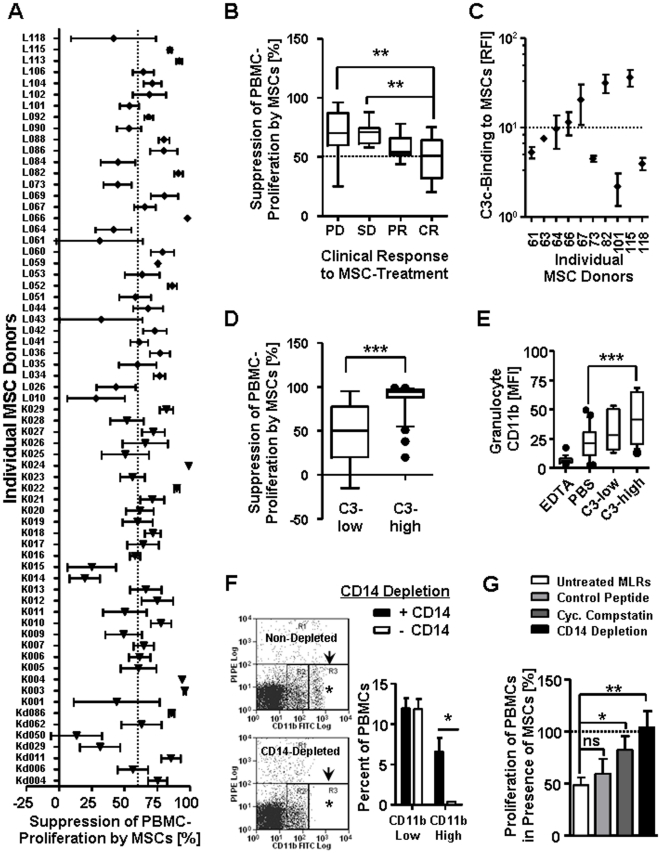
The complement activating properties of MSCs direct their interaction with complement receptor bearing effector cells and their immunomodulatory properties. (**A**) Suppression of PBMC-proliferation by MSCs was tested in MLRs. Retrospective analysis is shown (2003 – 2011), with an average of n = 5 MLR-experiments for each individual MSC-donor versus 3-5 random PBMC donors, the dotted line indicates overall average suppression (∼60%); (**B**) Average suppression of MSCs in MLRs and resulting clinical response to MSCs therapy (PD, progressive disease; SD, stable disease; PR, partial response; and CR, complete response); (**C**) MSC-donor specific differences in cell surface binding of C3-fragments after incubation with NHS as detected by binding of anti-C3c with flow cytometry (RFI, n>5 each donor), dotted line indicates threshold for C3-low (average RFI_C3c_<10) and C3-high cells (RFI_C3c_>10); (**D**) Statistical comparison for suppressive potency of MSCs in MLRs, when grouped into C3-low and C3-high cells (n = 17 each); (**E**) Flow cytometric analysis on CD11b-triggering activity of MSCs in human blood (MFI CD11b on PMNs), when compared for C3-low (n = 6) and C3-high cells (n = 14), relative to PBS- or EDTA-treated control blood. (**F**) MACS depletion of CD14+ cells from PBMCs efficiently removes of CD11b-high monocytes but not CD11b-low NK cells from MLRs (n = 5). (**G**) Suppression of PBMC-proliferation by MSCs in MLRs (n>3 experiments) with various combinations of MSCs (n = 5 donors), responder PBMCs (n = 2), and 2 different pools of allogeneic stimulator PBMCs (n = 5 donors), which were tested in the presence of different inhibitory treatments: untreated MLRs (n = 13, white), control peptide (linear Compstatin 20 µM, n = 13, light grey), cyclic Compstatin (20 µM, n = 13, dark grey), or with monocyte depletion (CD14/CD11b high cells, n = 9, black). The values in A-E are means±SEM, with *P<0.05, **P<0.01, and ***P<0.001.

Many clinical applications of MSCs are based on their immunomodulatory properties and tests to determine the potency of MSCs to elicit desired clinical responses would be anticipated. We therefore screened the suppressive effect of more than 60 individual MSCs in mixed lymphocyte reactions (MLRs), and found that MSCs show a broad donor specific variation in their suppressive properties, with an average inhibition of about 60% (Fig. 5A). Whilst some MSCs were highly suppressive in a consistent fashion (e.g. Kd086, K03, K22, K29, L82, L113), others showed strong variation (e.g. K01, L10, L43, L61, L118), some suppressed poorly (e.g. Kd029, KD050, K14, K15, L10), whereas others occasionally acted stimulatory in MLRs (e.g. Kd050, L43, L61). In an attempt to identify the most beneficial phenotype we correlated the average suppressive activity of MSCs in MLRs with their clinical response in patients.

Forty-two patients received 64 MSC infusions as experimental treatment of life-threatening complications to HSCT, with indication for ameliorating aGvHD and tissue damage. Patient characteristics are shown in table 1. No adverse events during or after MSC infusion were observed. Patients responded to 66% of infusions with 14 partial and 28 complete responses. We performed retrospective analysis of the clinical response to each individual MSC-infusion and found a weak inverse correlation with their average suppressive activity in MLRs (not shown). MSCs given to complete responders showed an average suppressive activity of ∼50% in MLRs, whereas non-responders had a significantly higher suppressive effect (P<0.0012, Figure 5B), which suggests that a strong suppressive activity of MSCs on PBMC-proliferation *in vitro* does not necessarily indicate a high therapeutical value for modulation of immune responses *in vivo*.

**Table 1:** Patients treated with MSCs.

Patients	
**Sex** (M/F)	30/12
**Age** median (range) child/adult	42 (1-67) 12/30
**Patient diagnosis:**	
Hematological malignancies	35
Solid tumor	3
Non-malignant disorders	4
**Patient conditioning:**	
MAC	26
RIC	16
**GvHD prophylaxis:**	
CsA+MTX	30
Other	12
**Indication for MSC treatment:**	
Graft-versus-host disease	28
Hemorrhagic cystitis	14
**Clinical response to treatment:**	28/42 (67%)
Complete response	20/42 (48%)
Partial response	08/42 (19%)
Stable disease	03/42 (07%)
Progressive disease	11/42 (26%)
**MSC donors**	
**Sex** (M/F)	16/17
**Age** median (range)	37 (1-66)
**Cell dose** median (range) (×10^6^/kg)	1.6 (0.6-3.0)
**Cell passage** median (range)	3.0 (1.0-4.0)
**Number of MSC infusions:**	64
**HLA-match with recipient:**	
3^rd^ party unrelated donor	50
Haploidentical related donor	11
HLA-identical sibling	3
**Clinical response to treatment:**	42/64 (66%)
Complete response	28/64 (44%)
Partial response	14/64 (22%)
Stable disease	08/64 (13%)
Progressive disease	14/64 (22%)

Abbreviations: MAC, myeloablative conditioning; RIC, reduced intensity conditioning; CsA, cyclosporine; MTX, methotrexate; and HLA, human leukocyte antigen.

To investigate weather the complement triggering properties of MSCs affect their immunomodulatory profile *in vitro* we repeatedly tested MSCs from 10 different cell donors; We quantified their average C3-fragment binding capacity and set a threshold (at RFI = 10), to distinguish between weakly or strongly complement activating cells (Fig. 5C). Highly C3 activating MSCs (C3-high, RFI_C3c_>10) showed to be far superior to C3-low cells (RFI_C3c_<10) in suppressing PBMC proliferation *in vitro* (P<0.001, Fig. 5D), and furthermore showed to be much more efficient in triggering of CD11b^+^-effector cells in whole blood (P<0.001, Fig. 5E). We consequently depleted the CD14^+^-cells from PBMCs, which lead to an efficient removal of CD14/CD11b-high myeloid effector cells from MLRs (P<0.05, Fig. 5F). The suppressive activity of MSCs was abrogated in monocyte depleted alloantigen-stimulated MLRs (P<0.01, Fig. 5G), and also significantly reduced after inhibition of complement at its central activation step C3 with Compstatin (P<0.05, Fig. 5G), but was not affected by its linear control peptide. Blocking of individual receptors downstream of C3, such as the C5a receptor with C5aR-antagonist, and complement receptor 3 (CD11b/CD18) with a CD11b blocking antibody was not sufficient to impair the suppressive activity of MSCs in MLRs (data not shown).

## Discussion

In recent years, the interaction of MSCs with the adoptive immune system has been extensively studied [27]. However, their relationship to the innate immune system has scarcely been addressed so far, focusing primarily on innate effector cells and toll like receptors [9], [27]. In the present study, we have investigated the interactions between culture-expanded MSCs and the complement system, which plays an important role in host defense, and in modifying immune responses *in vivo* [12], [13]. Here, we report for the first time that exposure of MSCs to complement-active human serum and blood leads to deposition of complement activation products on the cell surface of MSCs and generation of soluble anaphylatoxins. This process led to a complement-mediated triggering of effector cell activation, via the engagement of complement receptor type 3 (CD11b/CD18). The complement-activating properties of these cells were correlated with their immunomodulatory capacity to suppress PBMC-proliferation *in vitro*. The suppressive effect of MSCs could be blocked by inhibiting complement function or by removal of myeloid effector cells from MLRs. Thus, it appears that complement activation plays an important role in mediating the activation and interaction of MSCs with different types of complement receptor-bearing effector cells in human blood, potentially triggering their own intrinsic immunosuppressive functions and that of other effector cells, to generate a complex immunosuppressive environment (Figure S1).

It is generally believed that bone marrow derived MSCs migrate throughout the vascular system and home to specific target sites *in vivo*, but whether trafficking of MSCs occurs via the blood stream in healthy adults remains controversial [28]. We speculated that MSCs have a certain degree of intrinsic blood compatibility, such as attributed to endothelial cells (ECs), and studied how they interact with the complement system upon exposure to human serum. In our first set of experiments, we saw an enhanced cell surface C3-fragment deposition (iC3b and C3dg) and fluid phase generation of C3a after exposure of MSCs to human serum. Interestingly, Schraufstatter et al. recently demonstrated that complement anaphylatoxins C3a and C5a bind to their concomitant receptors on MSCs, which are then quickly translocated to the cell nucleus, where they trigger cell activation and chemotactic responses of these cells [15]. Furthermore, it was shown that complement modulates the inflammatory response of mesenchymal and more mature osteoblastic cells [29]. The activation of complement on the surface of culture expanded MSCs and the generation of anaphylatoxins in proximity to respective receptors on these cells may therefore provide a fast auto-activation loop, which could potentially trigger the immunosuppressive function of MSCs directly after systemic infusion. The requirement of this initial licensing step for MSC-function has already been well described for a number of pro-inflammatory cytokines such as interferon gamma [10]. Complement activation may therefore provide yet another powerful signal to activate the intrinsic defense mechanisms of MSCs after systemic infusion.

Cells in contact with blood are normally equipped to down-regulate complement activation via expression of complement regulatory proteins such as MCP, DAF, and CD59, which are typically found on ECs [30]. Culture-expanded MSCs lacked MCP and DAF, but were protected from complement-mediated lysis by the expression of CD59. We investigated potential complement-triggering factors and the specific profile of C3 fragments deposited onto the surface of MSCs, by using methods that paralleled those described in a previous study on islet cells [31]. Flow cytometry and western blot analysis showed cell-surface deposition of iC3b and C3dg on MSCs. Two typical mediators of complement activation are the recognition molecules C1q (classical pathway), which preferentially binds to antibodies, and MBL (lectin pathway), recognizing non-self carbohydrate ligands. Both cell types bound similar, but very low amounts of immunoglobulins. Scarce amounts of C1q and MBL were found to be primarily associated with non-vital cells, in agreement with the literature; typically, only late apoptotic or necrotic cells are recognized by these pattern recognition molecules [32].

To further investigate the biological potential of our findings, we used lepirudin anti-coagulated blood in order to perform more detailed studies on the interactions between MSCs and different types of blood effector cells without impairing complement function [20]. We found an enhanced generation of complement anaphylatoxins C3a and C5a, but no complement-mediated lysis of MSCs in blood. We also found activation of different types of blood effector cells, as indicated by triggering of CD11b-expression on PMNs and monocytes in response to MSCs. Compstatin was effective in blocking complement activation, which led to a reduction in C3 fragment deposition and anaphylatoxin generation. Both Compstatin and the C5aR antagonist successfully prevented the upregulation of CD11b on PMNs. Most importantly, both the degree of complement triggering elicited by MSCs and the resulting intensity of CD11b-mediated effector cell priming were correlated with the capacity of these cells to suppress the proliferation of PBMCs *in vitro*.

To understand the role of complement activation in this process we performed different blocking and cell depletion experiments and found that inhibition of complement at its central activation step C3 with Compstatin could diminish the suppressive effect or MSCs *in vitro*. This could not be achieved by inhibiting single downstream events of C3-activation, e.g. by blocking the C5a receptor or by blocking complement receptor 3. However, removal of CD14/CD11b-high myeloid effector cells (monocytes), strongly impaired the immunosuppressive function of MSCs *in vitro*, which is in agreement with earlier observations by Groh et al. who found that MSCs engage monocytes to elicit their immunosuppressive effects [33]. To clarify if a highly suppressive MSC-phenotype is actually associated with a beneficial clinical response *in vivo* we repeatedly tested the suppressive effect of clinical MSCs in alloantigen- and PHA-stimulated MLRs. We thereby obtained their average suppressive activity, which was then correlated with the degree of their individual clinical response obtained in treatment of acute GvHD and hemorrhagic cystitis. It appeared that MSCs with a medium suppressive activity are therapeutically more beneficial then highly MLR-suppressing MSCs, which might indicate that the strongly complement depositing phenotype is less favourable for therapeutical use.

A growing body of evidence suggests that the clinical infusion of MSCs can transform a pro-inflammatory environment into a milieu that favors healing and the suppression of allogeneic responses. This effect might be mediated by soluble factors and the generation of suppressive myeloid and lymphoid cell subsets. Many investigators have shown that MSCs inhibit the function of effector cells and may even polarize their phenotype to an immunosuppressive one, which possibly augments their own intrinsic immunosuppressive function [27]. Recent studies have pointed to the generation of suppressive myeloid cells after close interaction with MSCs, but the nature of this interaction was found to be elusive [8], [9]. This report shows that complement activation serves as a mediator between MSCs and complement receptor bearing immune cells, such as CD11b+-myloid and NK cells, and that complement activation augments the immunomodulatory activity of MSCs *in vitro*. Our findings also demonstrate that strongly complement activating MSCs might not essentially be more valuable for therapeutical use, since average suppressors appeared to yield the most beneficial therapeutical effect *in vivo*. Our results may contribute to the understanding and interpretation of complement mediated interactions of MSC with other immune cells *in vitro* and *in vivo*.

## Supporting Information

Figure S1 **The complement-activating properties of mesenchymal stem cells (MSCs).** Triggering of complement activation on the surface of MSCs leads to C3 convertase-mediated cleavage of complement factor C3 into its active fragments C3a and C3b. The covalently bound C3b can be degraded to iC3b by factor I. C3b and its degradation products mediate phagocytosis and immune responses via complement receptors, such as CR3 (CD11b/CD18), on host cells. Accumulation of C3b leads to assembly of C5 convertases that activate C5 to C5a and C5b, which may eventually lead to formation of the lytic membrane attack complex (MAC). However, cell lysis can be prevented by the complement regulatory function of membrane protein CD59. Anaphylatoxins C3a and C5a induce cell activation and chemotactic responses by binding to their receptors C3aR and C5aR on host cells and MSCs, which may promote interaction with various types of CR-bearing cells. Activated MSCs may reprogram host cells to synergistically produce an anti-inflammatory microenvironment composed of many different factors (iNOS/NO, IDO/kynurenine, HO-1/biliverdin and CO, PGE2, Galectin-1, TSG-6, sHLA-G5, HGF, IL6, IL10, TGFb, IL1Rag), and may suppress allogeneic immune responses in vivo.
(TIF)

Table S1 **MSC-induced effector cell activation in whole blood.** * Percentage (means±SD, n = 14) of resting or activated effector cells is shown for blood treated w/wo MSCs. *P<0.05, **P<0.01, and ***P<0.001 relative to non-MSC-treated blood.
(DOCX)
