# Supplementary figures and images for: Mesenchymal Stromal Cells Engage Complement and Complement Receptor Bearing Innate Effector Cells to Modulate Immune Responses
Source: PLoS One. 2011 Jul 1;6(7):e21703. doi: 10.1371/journal.pone.0021703 (PMC3128611; doi:10.1371/journal.pone.0021703)

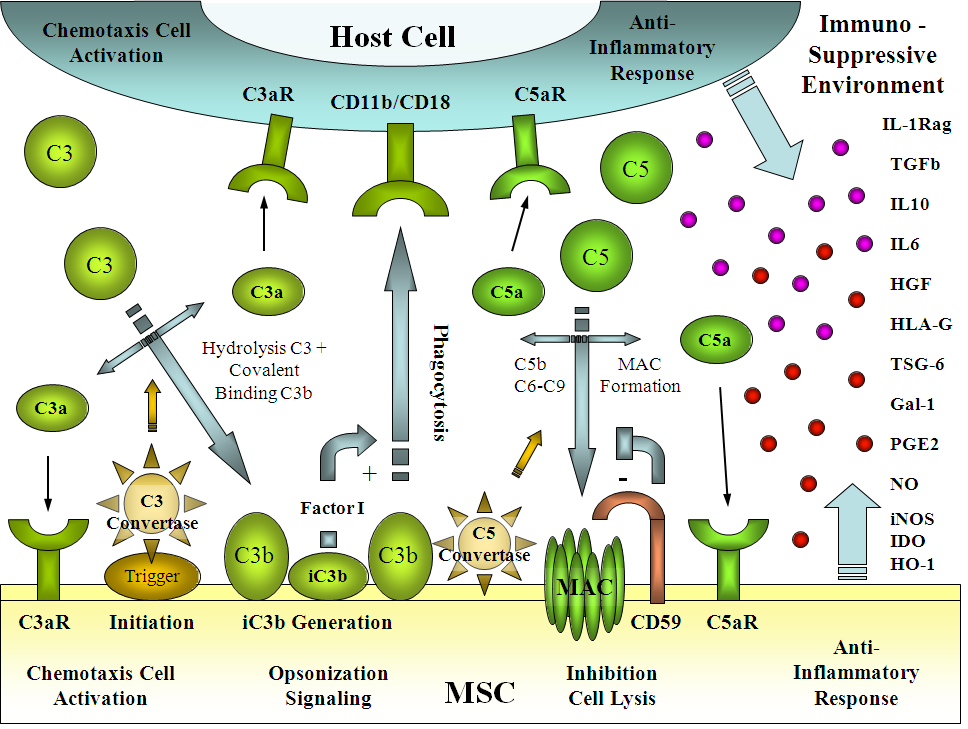

Supplement: Figure S1 — The complement-activating properties of mesenchymal stem cells (MSCs). Triggering of complement activation on the surface of MSCs leads to C3 convertase-mediated cleavage of complement factor C3 into its active fragments C3a and C3b. The covalently bound C3b can be degraded to iC3b by factor I. C3b and its degradation products mediate phagocytosis and immune responses via complement receptors, such as CR3 (CD11b/CD18), on host cells. Accumulation of C3b leads to assembly of C5 convertases that activate C5 to C5a and C5b, which may eventually lead to formation of the lytic membrane attack complex (MAC). However, cell lysis can be prevented by the complement regulatory function of membrane protein CD59. Anaphylatoxins C3a and C5a induce cell activation and chemotactic responses by binding to their receptors C3aR and C5aR on host cells and MSCs, which may promote interaction with various types of CR-bearing cells. Activated MSCs may reprogram host cells to synergistically produce an anti-inflammatory microenvironment composed of many different factors (iNOS/NO, IDO/kynurenine, HO-1/biliverdin and CO, PGE2, Galectin-1, TSG-6, sHLA-G5, HGF, IL6, IL10, TGFb, IL1Rag), and may suppress allogeneic immune responses in vivo. (TIF) [file pone.0021703.s001.tif]
