# Supplementary material for: Mesenchymal Stromal Cells Engage Complement and Complement Receptor Bearing Innate Effector Cells to Modulate Immune Responses
Source: PLoS One. 2011 Jul 1;6(7):e21703. doi: 10.1371/journal.pone.0021703 (PMC3128611; doi:10.1371/journal.pone.0021703)
Supplement: Table S1 — MSC-induced effector cell activation in whole blood. * Percentage (means±SD, n = 14) of resting or activated effector cells is shown for blood treated w/wo MSCs. *P<0.05, **P<0.01, and ***P<0.001 relative to non-MSC-treated blood. (DOCX) [file pone.0021703.s002.docx]

| **Type of** | **Effector cell activation in human blood** * | | | |
| --- | --- | --- | --- | --- |
| **effector cell** | **Resting PBMCs, %** | | **Activated PBMCs, %** | |
| **in blood** | **Without MSCs** | **With MSCs** | **Without MSCs** | **With MSCs** |
| **Monocytes** | 5.7 ± 2.0 | 3.8 ± 2.0*** | 2.6 ± 1.5 | 4.4 ± 1.9** |
| **Lymphocytes** | 32.2 ± 5.3 | 30.6 ± 5.1** | 1.7 ± 0.9 | 2.8 ± 1.3** |
| **Granulocytes** | 58.2 ± 8.2 | 56.7 ± 8.3* . | ND | ND |
| **Sum of PBMCs** | 96.1 ± 5.2 | 91.1 ± 5.1 . | 4.3 ± 1.2 | 7.2 ± 1.7 . |
